# Multifunctional AIE iridium (III) photosensitizer nanoparticles for two-photon-activated imaging and mitochondria targeting photodynamic therapy

**DOI:** 10.1186/s12951-021-01001-4

**Published:** 2021-08-23

**Authors:** Xuzi Cai, Kang-Nan Wang, Wen Ma, Yuanyuan Yang, Gui Chen, Huijiao Fu, Chunhui Cui, Zhiqiang Yu, Xuefeng Wang

**Affiliations:** 1 Department of Obstetrics and Gynecology, The Third Affiliated Hospital of Southern Medical University, Guangzhou, 510632 China; 2 Shunde Hospital, Southern Medical University (The First People’s Hospital of Shunde), Foshan, 528308 Guangdong China; 3 Department of Chemical and Biomolecular Engineering, National University of Singapore, 4 Engineering Drive 4, Singapore, 117585 Singapore; 4 Guangdong Provincial Key Laboratory of New Drug Screening, School of Pharmaceutical Sciences, Southern Medical University, Guangzhou, 510515 China; 5 Department of General Surgery, Zhujiang Hospital of Southern Medical University, Guangzhou, 510250 China

**Keywords:** Cyclometalated iridium nanoparticles, Two-photon excitation, Mitochondria-targeted, Fluorescence imaging, Photodynamic therapy

## Abstract

Developing novel photosensitizers for deep tissue imaging and efficient photodynamic therapy (PDT) remains a challenge because of the poor water solubility, low reactive oxygen species (ROS) generation efficiency, serve dark cytotoxicity, and weak absorption in the NIR region of conventional photosensitizers. Herein, cyclometalated iridium (III) complexes (Ir) with aggregation-induced emission (AIE) feature, high photoinduced ROS generation efficiency, two-photon excitation, and mitochondria-targeting capability were designed and further encapsulated into biocompatible nanoparticles (NPs). The Ir-NPs can be used to disturb redox homeostasis in vitro, result in mitochondrial dysfunction and cell apoptosis. Importantly, in vivo experiments demonstrated that the Ir-NPs presented obviously tumor-targeting ability, excellent antitumor effect, and low systematic dark-toxicity. Moreover, the Ir-NPs could serve as a two-photon imaging agent for deep tissue bioimaging with a penetration depth of up to 300 μm. This work presents a promising strategy for designing a clinical application of multifunctional Ir-NPs toward bioimaging and PDT.

**Supplementary Information:**

The online version contains supplementary material available at 10.1186/s12951-021-01001-4.

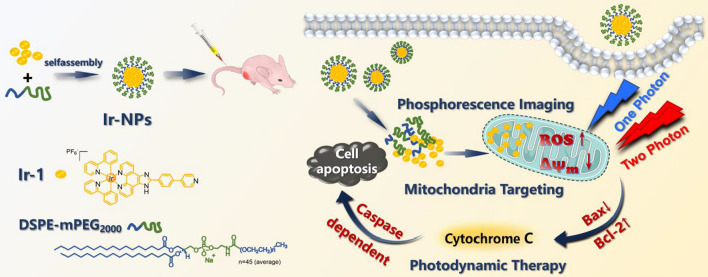

## Introduction

Photodynamic therapy (PDT), an emerging approach for oncotherapy, has possessed the advantages of reasonable specificity, non-invasiveness, and minimal side effects [1]. It generally depends on photosensitizers (PSs) to produce reactive oxygen species (ROS), such as singlet oxygen (^1^O_2_) and hydroxyl radicals (OH^·^) from ground-state O_2_ (^3^O_2_) inside tumor tissue, which would further lead to severe oxidative stress and then trigger cell death [2, 3]. Owing to the impressive photophysical properties, some of PSs can also be used as bioimaging agents. Hence, the PSs could be viewed as promising candidates for multifunctional theranostic agents combining PDT and imaging functionalities [4, 5].

However, the application of most PSs is restricted by their low water-solubility, poor selectivity, and high dark toxicity [6, 7]. Encapsulating PSs into water-dispersible polymeric nanoparticles (NPs) has been verified to be an attractive approach to increase the bioavailability, specificity, and biocompatibility of PSs [8]. Besides, organelle-targeted PSs have been widely reported to greatly enhance the treatment efficiency in the PDT process [9–11]. Unfortunately, as a result of aggregation in whether NPs or organelle, quenched fluorescence and decreased ROS production of conventional PSs would negatively impair PDT efficacy [12]. PSs with aggregation-induced emission (AIE) attributes exhibit increased fluorescence in the aggregated state, which could perfectly solve the difficulty in the clinical use of image-guided PDT for the conventional PSs [13–16]. As is known to all, in vivo optical imaging at near-infrared (NIR) wavelengths can both increase depths of biological tissues and effectively avoid the interference of autofluorescence from living tissues [17, 18]. Therefore, instead of one-photon absorption in the ultraviolet visible (UV–vis) region, two-photon absorption in the NIR region is a more competitive property in bioimaging [19]. However, developing novel two-photon PSs with AIE features for both deep tissue imaging and PDT therapy remains challenging.

In recent years, iridium (Ir) complexes have been regarded as ideal candidates for PSs to meet many basic requirements and offer significant advantages for PDT and applications [20–22]. In particular, cyclometalated Ir(III) complexes are considered outstanding probes for biological sensing and imaging due to their high phosphorescence quantum yield, excellent photostability, and large Stokes shift, etc. [23–27]. It is also recognized as a promising anticancer candidate owing to its excellent ^1^O_2_ quantum yield and sub-organelle targeting properties, which play a crucial role in mediating cell death, such as apoptosis, pyroptosis, and ferroptosis, etc. [28, 29]. Despite many good merits, Ir(III) complexes still have some drawbacks, such as limited water solubility, poor tumor-targeting capability and weak absorption in the NIR region, thus making it difficult to satisfy both therapeutic and imaging requirements.

Considering the above, cyclometalated Ir(III) complexes with the advantages of high ^1^O_2_ quantum yield, two-photon excitation, AIE characteristics, and mitochondria-targetability were designed, synthesized, and further encapsulated into NPs. The results showed that Ir-NPs were taken up by the Skov3 cell line, and the Ir(III) complexes were mainly localized in mitochondria and able to generate ROS under white light irradiation. Subsequently, the excessive ROS resulted in mitochondrial dysfunction and induced cell apoptosis. Furthermore, the great antitumor efficacy with little side effects and excellent two-photon bioimaging of tissues make these Ir-NPs an attractive candidate for cancer theranostics.

## Results and discussion

### Synthesis and characterization of Ir complexes

**Fig. 1:**
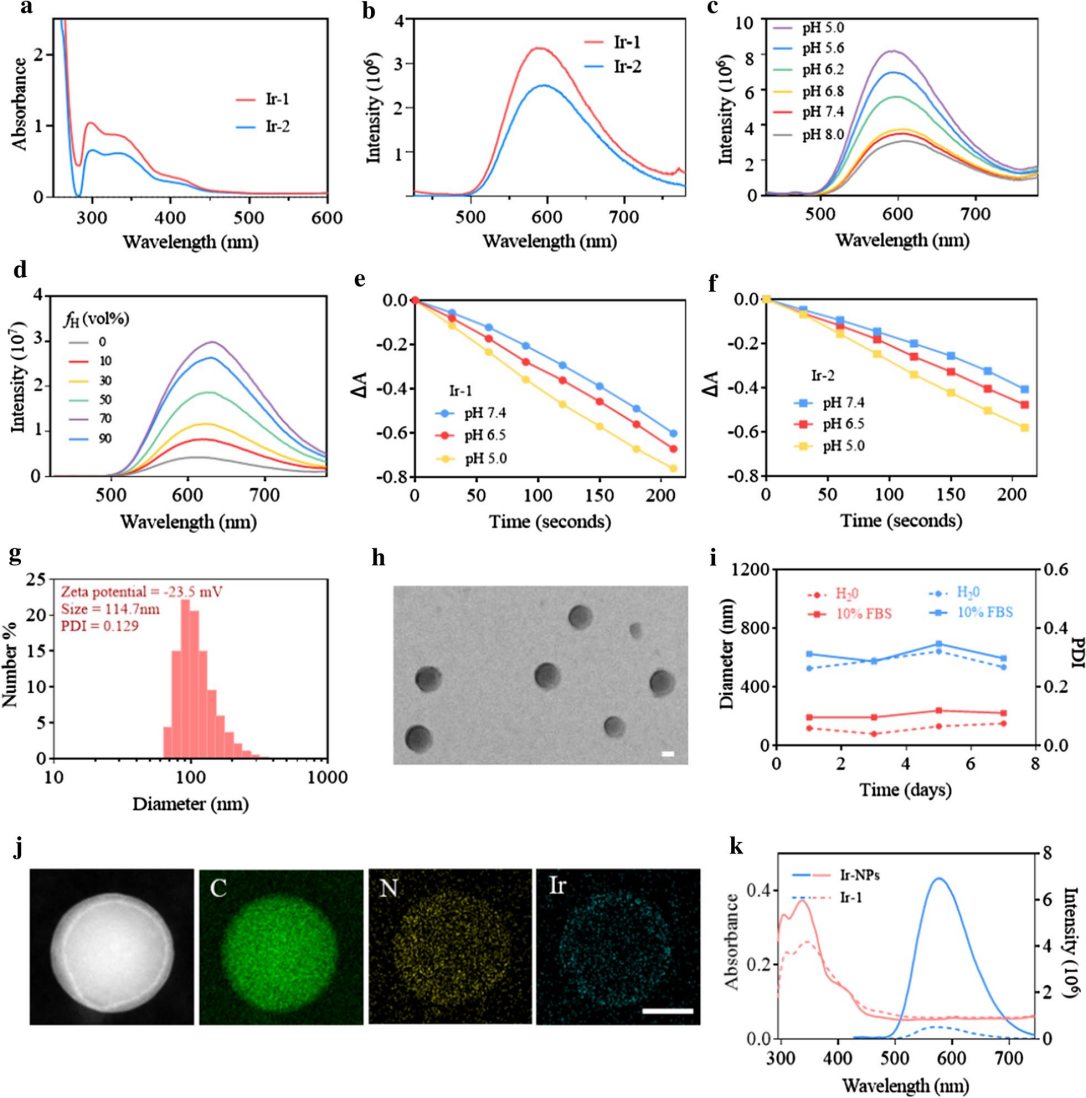
Photophysical properties of Ir complexes and characteristics of Ir-NPs. The absorption (**a**) and phosphorescence spectra (**b**) of Ir complexes (10 μM) in H_2_O. pH-sensitive emission spectra of Ir-1 in disodium hydrogen phosphate-citric acid buffer solution (**c**). Phosphorescence spectra of Ir-1 in H_2_O/DMSO mixtures with different H_2_O fractions (*f*_H_) (**d**). The decomposition rate of ABDA (100 μM) for Ir-1 (10 μM) (**a**) and Ir-2 (10 μM) (**b**) at pH 7.4, 6.5 and 5.0 under white light irradiation (50 mW/cm^2^) (**e**, **f**). The DLS data (**g**) and TEM image (**h**) of Ir-NPs, scale bar: 50 μm. The stability of Ir-NPs in PBS and RPIM 1640 culture medium with 10% FBS (**i**). The elemental mapping analysis of Ir-NPs (**j**), scale bar: 50 μm. The absorption and PL spectra of Ir-1 and Ir-NPs (5 μM) (**k**)

The two Ir complexes were designed and synthesized via a route shown in Additional file 1: Scheme S1. The chemical structure characterizations (^1^H NMR and ^13^C NMR) of the Ir-1 and Ir-2 are described in Additional file 1: Figures S1–S4. The results revealed the high purity and right structure of the Ir complexes. The optical absorption and phosphorescence emission spectra of Ir-1 and Ir-2 in H_2_O were investigated, respectively. Both Ir-1 and Ir-2 yielded intense absorption bands at 250–420 nm (Fig. 1a). Upon the excitation at 405 nm, the Ir complexes exhibited red phosphorescence emission with a peak at approximately 590 nm (Fig. 1b). The large Stokes shifts of the Ir complexes (approximately 185 nm) could avoid cross-talk during fluorescence imaging. The phosphorescence emission intensities of the Ir complexes responded to pH changes, with clear increases from pH 8.0 to 5.0 (Fig. 1c and Additional file 1: Fig. S5). Subsequently, the AIE property of both Ir complexes was characterized in H_2_O/DMSO mixtures at different ratios. Notably, the phosphorescence emission intensities of Ir-1 and Ir-2 were increased upon aggregation formation, exhibiting red emission in an AIE-active manner (Fig. 1d and Additional file 1: Fig. S6).

Besides, ^1^O_2_ generation of the two Ir complexes was evaluated with the use of 9,10-anthracenediylbis(methylene)-dimalonic acid (ABDA) as an indicator. Compared with the standard used for ROS yield of metal complex [Ru(bpy)_3_]Cl_2_ (*Φ*_Δ_ = 0.18) [30], Ir-1 and Ir-2 have a higher ROS yield at pH 7.4, with investigated as 0.64 and 0.51, respectively (Fig. 1e). Furthermore, the ^1^O_2_ quantum yields of the Ir complexes was enhanced as the pH decreased (Fig. 1f). The highly efficient ROS generation of the Ir complexes in an acidic environment provided convenience to kill cancer cells within the acidic tumor microenvironment (pH 6.5–6.8). By comparison, Ir-1 has a better photosensitizing capability and more excellent responsiveness. Thus, it was chosen as the object of further research.

### Preparation and characterization of Ir-NPs

**Scheme 1.**
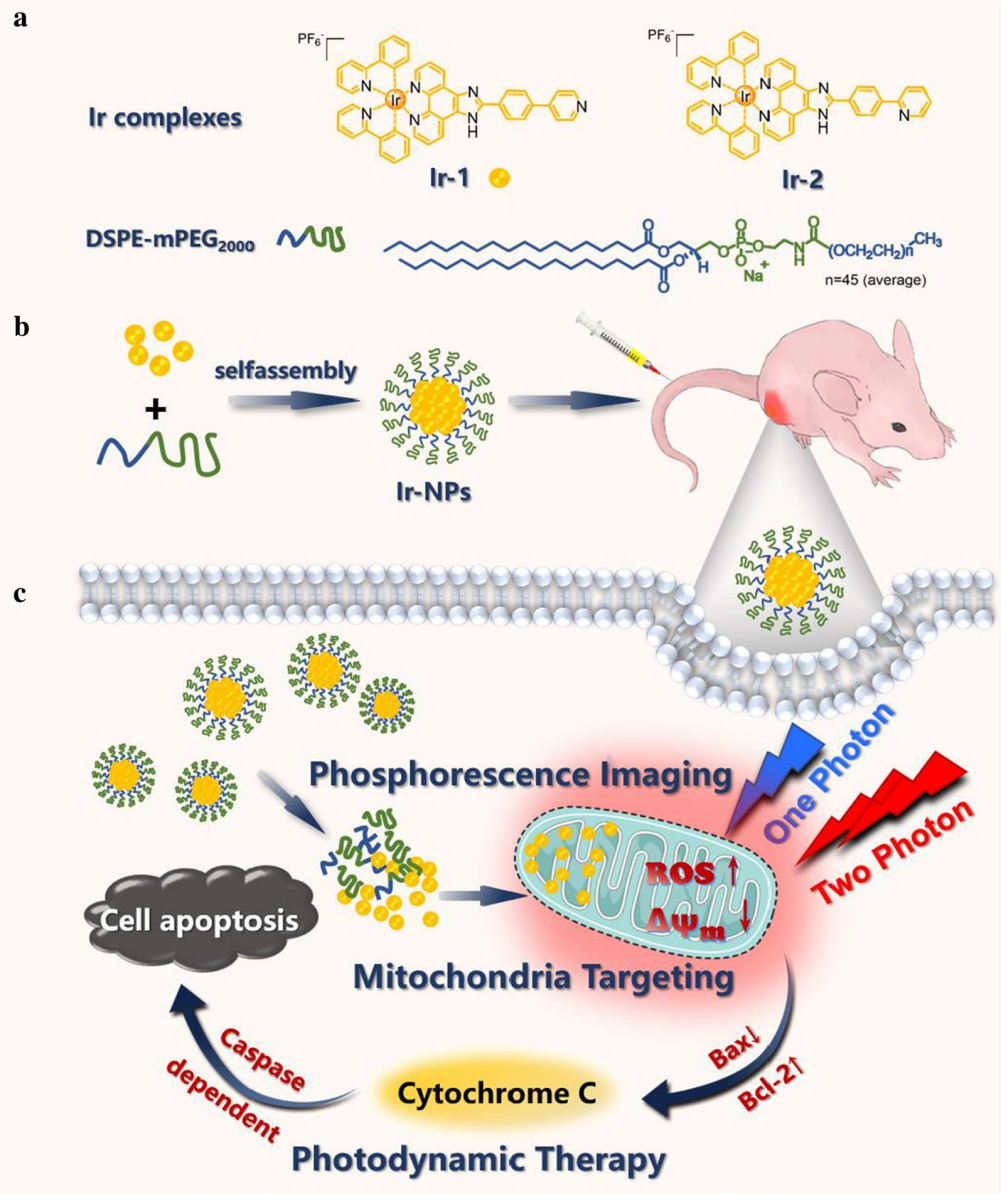
Schematic of the preparation of Ir-NPs for two-photon-activated phosphorescence imaging and mitochondria targeting photodynamic therapy

To improve the bioavailability and biocompatibility, Ir-1-encapsulated DSPE-mPEG_2000_ nanoparticles (Ir-NPs) were formed via nanoprecipitation method (Scheme 1) [31]. The dynamic light scatting (DLS) analysis (Fig. 1g) and transmission electron microscopy (TEM) images (Fig. 1h) revealed spherically shaped, monodispersed, and negatively charged Ir-NPs with a particle size of 114.7 nm (PDI < 0.3) were successful synthesized. The elemental mapping detected by TEM revealed the uniform distribution of carbon, nitrogen, and iridium in the nanoparticles (Fig. 1j and Additional file 1: Fig. S7). The stability of Ir-NPs was then investigated by DLS. The results showed that the Ir-NPs were stable in PBS and culture solution, as indicated by the negligible increase in the particle size at different time points for 7 days (Fig. 1i). Good stability is favorable for preventing drug leakage prematurely from Ir-NPs in physiological environment during blood circulation. Meanwhile, the appropriate size is beneficial for the efficient accumulation of Ir-NPs at the tumor sites owing to the enhanced permeability and retention (EPR) effect in vivo. Furthermore, the ROS yield, absorption and emission spectra of free Ir-1 and Ir-NPs in H_2_O were measured (Additional file 1: Fig. S8, Fig. 1k). The ROS yield of Ir-NPs was similar with Ir-1 which indicated that the NP carrier did not affect the photosensitizing capability of Ir-1. Notably, the emissive intensity of NP formation was much higher than that of free formation (by 13.5 times) because of the AIE property, in this sense, it would be more advantageous for the fluorescence imaging.

### Cellular imaging, localization, and internalization

**Fig. 2:**
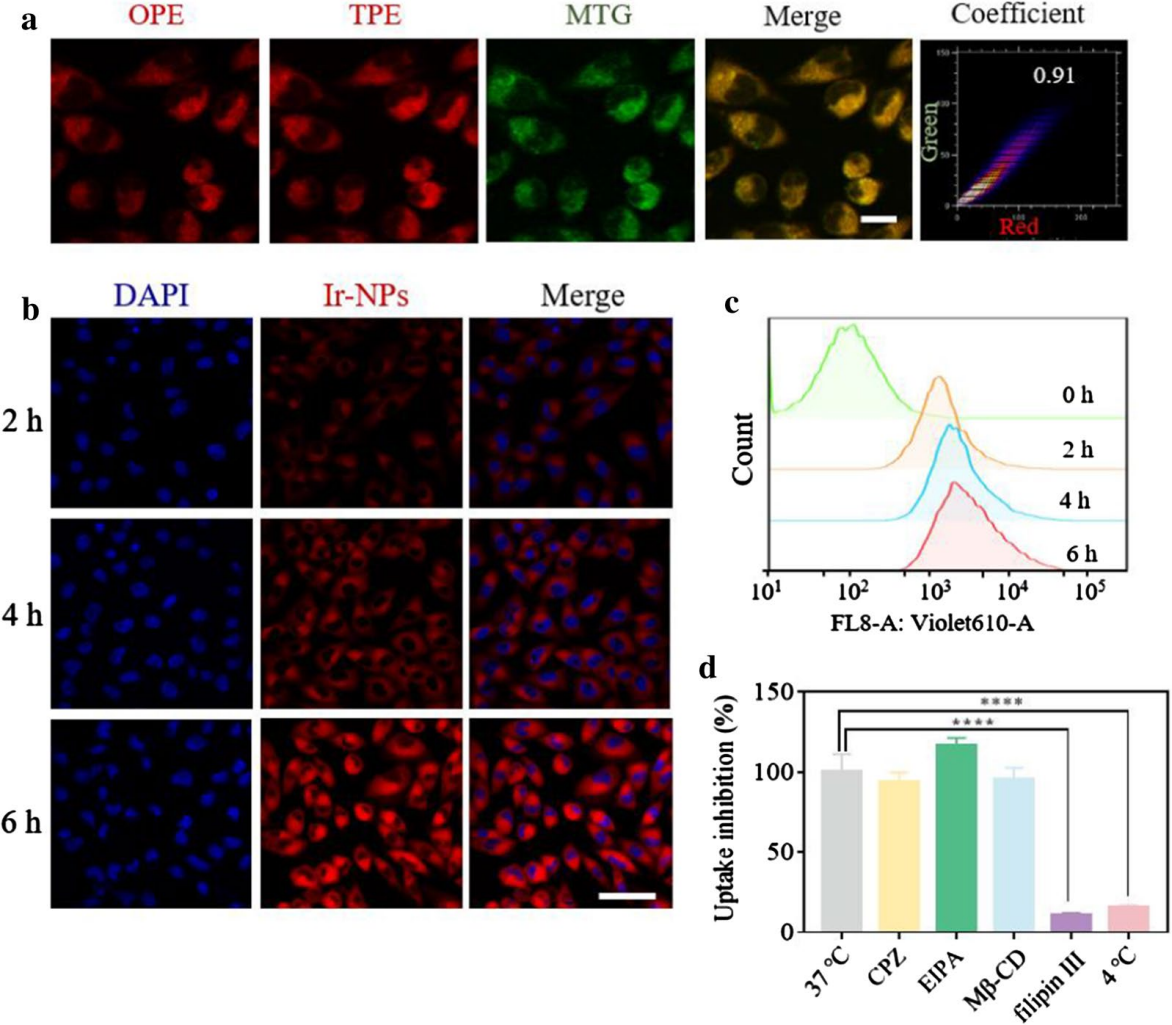
Cellular imaging, localization, and uptake. The OPE/TPE confocal images (**a**) of Ir-NPs distribution in Skov3 cells, scale bar: 10 μm. CLSM observation (**b**) and flow cytometer (**c**) of Ir-NPs uptake by Skov3 cells for 2, 4, and 6 h, respectively, scale bar: 50 μm. The uptake inhibition percent analysis (**d**) of Ir-NPs uptake by Skov3 cells upon different treatments of endocytosis inhibitors (CPZ, EIPA, Mβ-CD, and filipin III) at 37 °C and 4 °C, ****P < 0.0001. Ir-1: λ_ex_ = 405 nm (OPE)/810 nm (TPE), λ_em_ = 590 ± 20 nm, DAPI: λ_ex_ = 405 nm, λ_em_ = 440 ± 20 nm Mito-tracker Green (MTG): λ_ex_ = 488 nm, λ_em_ = 510 ± 20 nm

Subsequently, the cellular imaging capability, subcellular localization, and uptake pathway of Ir-NPs were investigated. As shown in Fig. 2a, after incubation in Skov3 cells, both one-photon excitation (OPE, λ_ex_ = 405 nm) and two-photon excitation (TPE, λ_ex_ = 810 nm) can present intense red phosphorescent signal from the Ir-NPs by confocal laser scanning microscope (CLSM) (Fig. 2a). Both CLSM observations and flow cytometry quantitative analyses indicated gradual internalization of Ir-NPs in Skov3 cells in a time-dependent manner (Fig. 2b, c, and Additional file 1: Fig. S9). The specific cellular uptake pathway of Ir-NPs was studied by pretreating cells with various endocytosis inhibitors or by incubating them at low temperatures. The pathways of clathrin-mediated endocytosis, micropinocytosis, lipid raft-dependent endocytosis, and caveolae-mediated endocytosis were inhibited by chlorpromazine (CPZ), ethylisopropylamiloride (EIPA), methyl-β-cyclodextrin (Mβ-CD), and filipin III, respectively [32]. Only the addition of filipin III resulted in a significant decrease in fluorescence (reduced to 10.4%) (Fig. 2d and Additional file 1: Fig. S10), thus indicating the Ir-NPs were taken up by Skov3 cells mainly through caveolae-mediated endocytosis. In addition, the uptake efficiency was markedly blocked when cells were incubated at 4 ℃ (reduced to 15.4%), which suggested that the uptake was an energy-dependent process. After the Ir-NPs were taken into the cells, the phospholipid component of the NP carrier DSPE-mPEG_2000_ could be easily decomposed by intracellular esterase [33], which would result in the release of Ir-1. Considering the structure of the lipophilic cations, Ir-1 was expected to target mitochondria in the cytoplasm. Therefore, the Mito-Tracker Green (MTG), a commercial mitochondrial fluorescent probe, was used to further determine the subcellular localization of Ir-1. As a result, a high level of colocalization was observed with a Pearson’s correlation efficient of up to 0.90 (Fig. 2a and Additional file 1: Fig. S11). Mitochondria are one of the most important cellular organelles for various vital physiological processes in organisms, including redox status maintenance, molecular metabolism and energy supply [34]. Thus, the property of mitochondrial targeting would help the nanomaterials directly damage mitochondria and maximize the cytotoxic effects of ROS.

### In vitro PDT performance

**Fig. 3:**
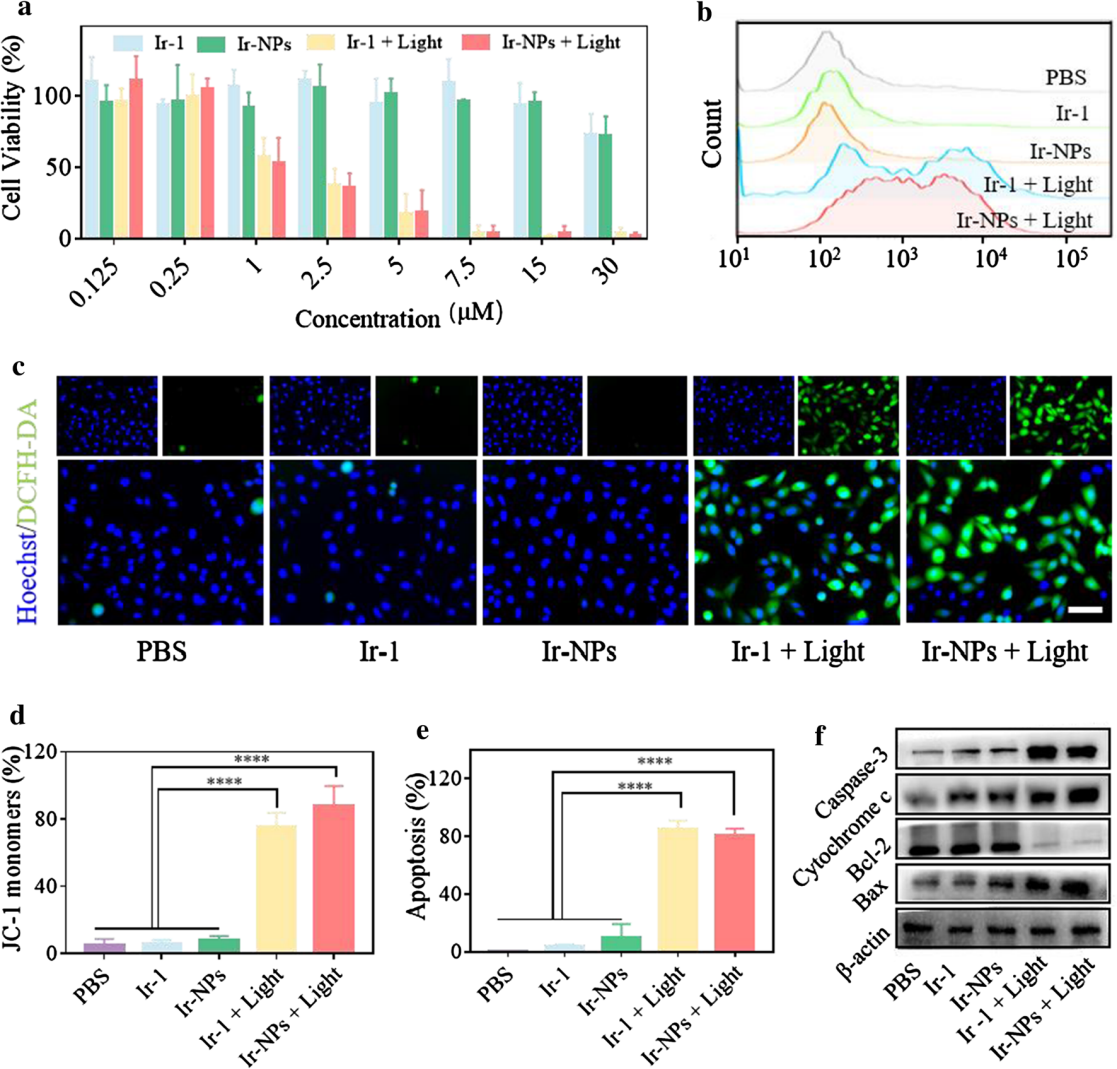
Cytotoxicity and mechanism evaluation of Ir-NPs in vitro. Cell viability (**a**) of Skov3 cells incubated with Ir-1 and Ir-NPs for 12 h with or without white light irradiation. ROS intensity in Skov3 cells after various treatments examined by flow cytometer (**b**) and CLSM observation (**c**), scale bar = 100 μm. Flow cytometer analysis of mitochondrial membrane potential (**d**) (using JC-1 as an indicator) for Skov3 incubated with Ir-1 and Ir-NPs for 12 h with or without white light exposure. Cell apoptosis level (**e**) of Skov3 cells incubated with Ir-1 or Ir-NPs for 12 h under white light irradiation or dark conditions, ****P < 0.0001. Western blot analysis (**f**) of mitochondrial apoptosis-related proteins in Skov3 cell after various treatments with or without white light irradiation

The cytotoxicity of Ir-1 and Ir-NPs in vitro under dark or white light irradiation (400–700 nm, 50 mW/cm^2^ for 5 min) conditions was examined with the MTT assay. Both free Ir-1 and Ir-NPs performed negligible toxicity to Skov3 cells in the dark, but exhibited strong cytotoxicity upon white light irradiation with the IC_50_ of 1.59 ± 0.18 μM and 1.24 ± 0.10 μM, respectively (Fig. 3a). These finding hinted to good biocompatibility and excellent phototoxicity of Ir-1 and Ir-NPs. CLSM observation with Calcein-AM/PI staining was further used to confirm the cell-killing efficacy of Ir-1 and Ir-NPs (Additional file 1: Fig. S12) upon white light irradiation, whose results were in conformity to the cytotoxicity results.

Under normal conditions, the level of ROS around mitochondria is in dynamic equilibrium, and the ROS overload would cause the mitochondrial membrane potential (MMP) to collapse, and further lead to mitochondrial damage and cell apoptosis [35]. Based on the efficient ROS generation ability in buffer solution and mitochondrial targeting characteristic of Ir-1, intracellular ROS levels were detected by 2ʹ,7ʹ-dichlorofluorescin diacetate (DCFH-DA) staining and analyzed by flow cytometry and CLSM. As shown in Fig. 3b, c, negligible ROS production was observed in Skov3 cells treated with Ir-1 or Ir-NPs in dark condition. While, once irradiation was conducted, the ROS production induced by Ir-1 and Ir-NPs was dramatically increased. MMP collapse is a typical characteristic of mitochondrial damage [36]. Therefore, MMP changes were monitored using the membrane-permeable JC-1 dye [37]. A decrease in red fluorescence (JC-1 aggregates) and an increase in green fluorescence (JC-1 monomers) were used to characterize mitochondrial depolarization. As shown in Additional file 1: Fig. S13 and Fig. 3d, after white light irradiation, the percentage of cells with MMP lose increase from 6.15 ± 1.94% to 75.45 ± 8.42% and 8.143 ± 5.95% to 87.75 ± 11.95% for Ir-1 and Ir-NPs, respectively. MMP collapse affects mitochondrial permeability and results in cytochrome *c* leakage, which further activates caspase-dependent apoptosis [38]. Bcl-2/Bax family proteins regulate the release of mitochondrial cytochrome *c* [36]. Bcl-2 acts as an anti-apoptotic factor via preventing cytochrome *c* release and maintaining outer mitochondrial membrane impermeability. In contrast, Bax induces mitochondrial cytochrome *c* release to promote cell apoptosis [36]. In the present study, the cell apoptosis induced by Ir-1 and Ir-NPs upon irradiation was verified by flow cytometry with Annexin V-FITC/PI staining (Fig. 1e and Additional file 1: Fig. S14). Moreover, the suppression of Bcl-2, increase of cytochrome *c*, activation of Bax and caspase-3 were observed in Skov3 cells treated with Ir-1 and Ir-NPs upon irradiation by western blotting (Fig. 3f and Additional file 1: Fig. S15). These results demonstrate that the PDT effects induced by Ir-1 or Ir-NPs disturbed redox homeostasis, gradually resulted in distinct mitochondrial dysfunction, and finally leaded to caspase-dependent cell apoptosis in Skov3 cells.

### In vivo biodistribution and bioimaging

**Fig. 4:**
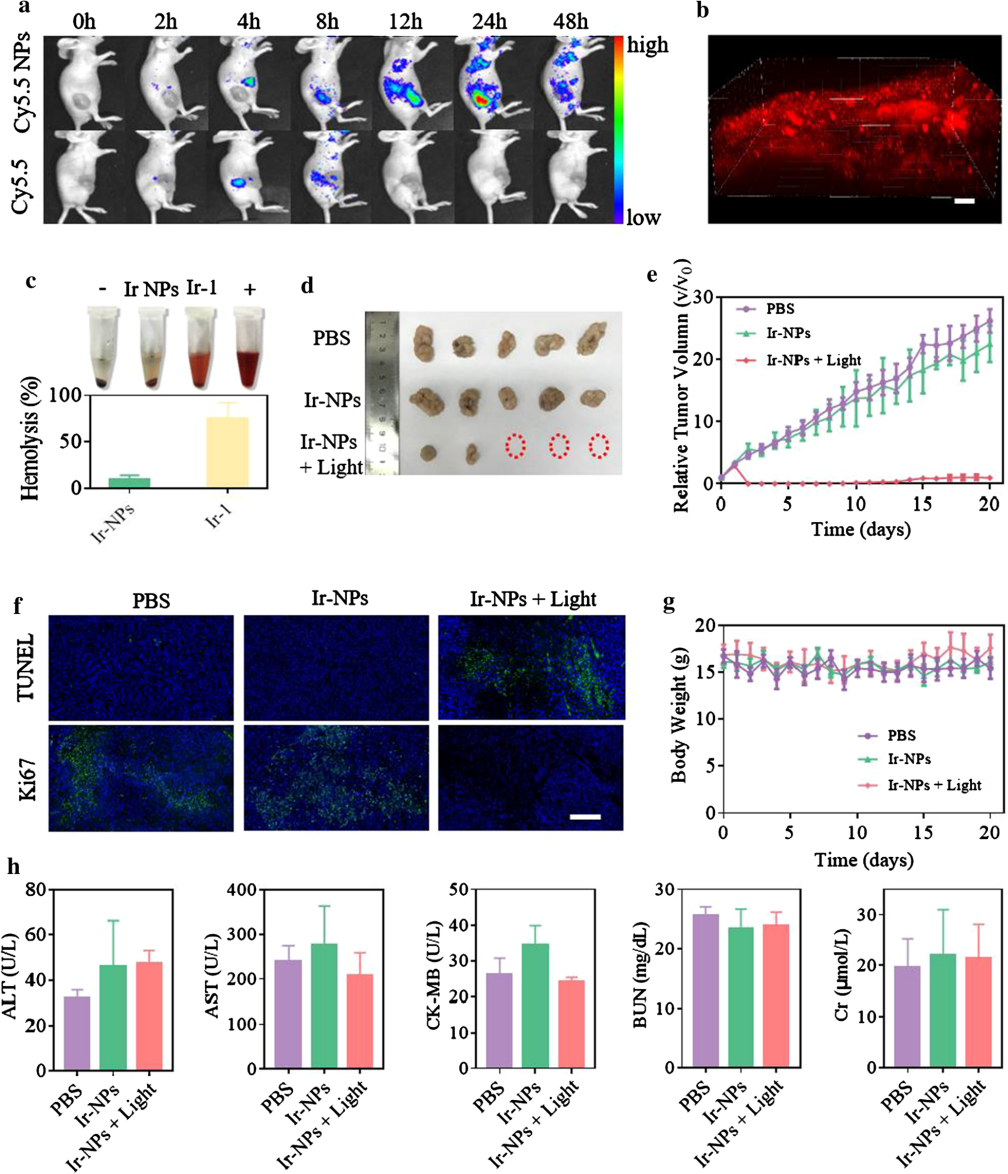
In vivo imaging and therapeutic efficacy of Ir-NPs. In vivo fluorescence biodistribution (**a**) of Cy5.5 NPs and free Cy5.5 in Skov3 tumor-bearing mice. 3D fluorescence imaging (**b**) of tumor tissue under two-photon excitation, λ_ex_ = 810 nm, λ_em_ = 590 ± 20 nm, scale bar: 100 μm. Hemolytic activity of Ir-1 and Ir-NPs (n = 3) (**c**). Image of the Skov3 tumors (**d**) isolated from mice after treatments of PBS, Ir-NPs (0.15 mg/kg) and Ir-NPs + Light (0.15 mg/kg). Tumor growth curves (**e**) and body weight (**f**) of Skov3 tumor-bearing mice during the therapeutic period (n = 5). **g** TUNEL assay and Ki67 immunofluorescence analysis of tumor sections after treatments, scale bar = 200 μm. **h** In vivo biological safety assessed by serum biochemical analysis after the treatment course (n = 3)

The encouraging in vitro antitumor efficacy inspired us to explore the biodistribution of the NPs in nude mice bearing subcutaneous Skov3 cells. DSPE-mPEG_2000_ is one of the most widely applicated polymer materials in long-circulating nanoparticles preparation and the nanoparticles formed with DSPE-mPEG_2000_ were reported to have the ability of tumor-specific accumulation in vivo due to the EPR effect [39]. To verify this conclusion, Cy5.5 was encased to form Cy5.5 NPs to access the biodistribution of DSPE-mPEG_2000_ nanoparticles, because the Cy5.5 has been extensively applied for nanoparticle tracing in biomedical fields owing to its merits of high molar absorption coefficient and fluorescence quantum yield [40]. After intravenous injection of Cy5.5 NPs or free Cy5.5 into tumor-bearing mice, the vivo images showed the clear fluorescence signal of Cy5.5. Owing to the benefit of passive targeting ability, the nanoparticles accumulated at the tumor site reached the maximum at 24 h, and remained strong at 48 h, while the signal of free Cy5.5 became negligible after 8 h post-injection (Fig. 4a). The ex vivo images and corresponding quantification (Additional file 1: Fig. S16) showed that the Cy5.5 NPs were mainly located in the tumor and liver at 24 h, while the free Cy5.5 was already metabolized. These results confirmed that the good long-circulation and tumor-specific accumulation of NPs. Owings to its excellent two-photon cellular imaging capabilities, tissue imaging was performed to estimate the bioimaging ability of Ir-NPs. After intravenous injection of Ir-NPs for 24 h, the tumors were harvested. As shown in Fig. 4b, a high-quality image of the tumor tissue was obtained by two-photon excitation at 810 nm, and the imaging depth reached up to 300 μm. Thus, the Ir-NPs were suitable for tumor bioimaging owing to their accumulation behavior at the tumor site, two-photon NIR excitation, high penetration depth, and high-quality images.

### In vivo PDT therapy

Considering the aggregation of red blood cells (RBCs) that positively charged Ir-1 may produce in the venous circulation in vivo, a hemolysis assay was conducted to evaluate the hemolytic activity of Ir-1 and Ir-NPs. With PBS and Triton X as the positive and negative control, the hemolytic activities of free Ir-1 and Ir-NPs were observed to be 74.8 ± 17.7% and 9.9 ± 4.2%, respectively (Fig. 4c). These results indicated that the free Ir-1 leads to a prominent hemolytic toxicity in mouse RBCs, while the Ir-NP coatings yield a considerably smaller effect. Thus, when the tumor volume grew to approximately 100 mm^3^, Skov3 tumor-bearing nude mice were randomly divided into three groups and received treatments of PBS, Ir-NPs under dark conditions (Ir-NPs), and Ir-NPs under white light irradiation (24 h post-injection, 400–700 nm, 200 mW/cm^2^ for 5 min) (Ir-NPs + Light). The therapeutic effect was evaluated by monitoring tumor volumes over a period of 3 weeks. As shown in Fig. 4d, e, the tumor volumes in the PBS and Ir-NPs groups increased by 26.2-fold and 22.5-fold respectively. However, in the Ir-NPs + Light group, the tumor growth was inhibited considerably, and over half of the mice tumors were ablated. Extensive shrinkage, fragmentation, and disappearance of nuclei were observed in H&E staining tumor tissues of Ir-NPs + Light group (Additional file 1: Fig. S17). Meanwhile, the results of the TUNEL assay and Ki67 immunofluorescence analysis illustrated that apoptosis was induced and proliferation was inhibited in the tumors of Ir-NPs + Light group (Fig. 4f). In addition, there was no significant difference in the body weights of mice in each group (Fig. 4h). To evaluate further the biological safety of Ir-NPs, H&E staining of major organs (heart, liver, spleen, lung, and kidney) and biochemical analysis of serum (AST, ALT, CK-MB, BUN, and Cr) were performed after the treatment course. No pathological or biochemical changes were observed in mice who received different treatments (Additional file 1: Fig. S18 and Fig. 4h). Taken together, these results indicate an excellent PDT effect and negligible systemic toxicity of Ir-NPs in Skov3 tumor-bearing mice.

## Conclusion

In summary, Ir-1 with merits of AIE, good photosensitivity, pH responsiveness, two-photon activated phosphorescence imaging and mitochondria-targeting capability was designed and synthesized. Additional attributes included its good bioavailability, biocompatibility, and tumor-targetability characteristics following its encapsulation into NPs. In vitro experiments showed that Ir-NPs disturbed redox homeostasis, resulted in mitochondrial dysfunction, and cell apoptosis in Skov3 cells. Moreover, Ir-NPs exhibited an impressive two-photon imaging performance. Importantly, in vivo experiments demonstrated that Ir-NPs had a good tumor-targeting ability, excellent antitumor effects, and low systematic toxicity. Therefore, this work presents a promising strategy for designing a clinical application of multifunctional Ir-NPs for bioimaging and PDT.

## Methods

### Materials and instruments

All solvents (analytical grade) and reagents were used as received from commercial sources unless otherwise indicated. Solvents were purified by standard procedures. 1,10-Phenanthroline, 4-Phenylpyridine, 2-Phenylpyridine, and IrCl_3_·nH_2_O were obtained as analytical reagents from Shanghai Reagents. 4-(2-Pyridinyl)benzaldehyde was purchased from Energy Chemical. Above materials were used as received directly without further purification. Disodium hydrogen phosphate, citric acid, tris aqueous solution (2-amino-2-hydroxymethylpropane-1,3-diol), Cy5.5 (sulfo-cyanine5.5 carboxylic acid), ABDA, DCFH-DA, phosphate buffered saline, 3-(4,5-dimethyl-2-thiazolyl)-2,5-diphenyl-2-H-tetrazolium bromide (MTT) and 4',6-diamidino-2-phenylindole (DAPI) were purchased from Aladdin (China). Annexin V-FITC/PI apoptosis detection kit, Calcein-AM/PI Double Stain Kit, JC-1-Mitochondrial Membrane Potential Assay Kit, bicinchoninic acid (BCA) protein assay kit, and goat anti-rabbit IgG antibody were purchased from Beyotime Institute of Biotechnology (China). RPMI 1640 medium, fetal bovine serum (FBS), and trypsin were purchased from Gibco BRL (USA). All antibodies were purchased from Abcam.

^1^H NMR and ^13^C NMR spectra were measured by a 400/500 MHz NMR spectrometer (Bruker). The morphology and size were measured by TEM (FEI talosf200s). The particle size distribution was measured by DLS analysis (Malvern Nano ZS90). The absorbance spectra and fluorescence spectra were measured using an UV–vis spectrometer (Varian Cary 300) and fluorescence spectrometer (Edinburgh FLS 920), respectively. The confocal images were recorded using multi-photon laser scanning microscopy (FV1200MPE).

### Synthesis and characterization of Ir complexes

Ir complexes of Ir-1 and Ir-2 were prepared according to the method reported in the reference [41]. The synthetic routes are depicted in Additional file 1: Scheme S1.

### General synthetic of Ir-1 and Ir-2

#### [Ir(ppy)_2_(Hppip-4)]PF_6_ (Ir-1)

A mixture of [Ir(ppy)_2_Cl]_2_ (0.20 mmol, 0.21 g) and Hppip-4 (0.41 mmol, 0.153 g) were dissolved in a flask with CH_2_Cl_2_/MeOH (2:1, v/v, 54 mL). Then, in the opaque background, the mixed solution was refluxed at 65 ℃ for 5 h under N_2_. After cooling to room temperature, NH_4_PF_6_ (0.3 g) was added and stirred for 1–2 h. The solution was filtered and the solvent of colatuie was removed by rotary evaporation. The resulting crude product was redissolved with CH_2_Cl_2_ and filtered. After recrystallization from diethyl ether, an orange product with a yield of 74% was obtained. [Ir(ppy)_2_(Hppip-2)]PF_6_ (Ir-2) was synthetic according to the method of Ir-1. The complexes were fully characterized by ^1^H NMR and ^13^C NMR to reveal the right structure and high purity of both compounds.

#### Ir-1

^1^H NMR (400 MHz, DMSO) δ 14.48 (s, 1H), 9.23 (t, *J* = 8.4 Hz, 2H), 8.72 (s, 2H), 8.48 (d, *J* = 8.4 Hz, 2H), 8.28 (d, *J* = 8.2 Hz, 2H), 8.22–8.09 (m, 6H), 7.97 (d, *J* = 7.2 Hz, 2H), 7.88 (dd, *J* = 15.2, 6.9 Hz, 4H), 7.53 (t, *J* = 6.4 Hz, 2H), 7.08 (t, *J* = 7.5 Hz, 2H), 6.99 (dt, *J* = 14.7, 7.2 Hz, 4H), 6.31 (d, *J* = 7.2 Hz, 2H), 5.76 (s, 1H). ^13^C NMR (101 MHz, DMSO) δ 167.38 (s), 152.51 (s), 150.84 (s), 149.63 (s), 148.95 (s), 146.41 (s), 144.72 (s), 144.50 (s), 139.16 (s), 132.73 (s), 131.69 (s), 130.74 (s), 130.50 (s), 128.12 (s), 127.63 (d, *J* = 11.2 Hz), 125.54 (s), 124.30 (s), 122.85 (s), 121.62 (s), 120.45 (s).

#### Ir-2

^1^H NMR (DMSO-d6, 500 MHz): 6.32 (d, *J* = 7.4 Hz, 2H), 6.96 (t, *J* = 7.5 Hz, 2H), 7.01 (t, *J* = 6.8 Hz, 2H), 7.06 (d, *J* = 7.5 Hz, 2H), 7.38–7.40 (m, 1H), 7.51 (d, *J* = 5.7 Hz, 2H), 7.87 (t, *J* = 8.2 Hz, 2H), 7.91–8.00 (m, 5H), 8.06–8.07 (m, 3H), 8.27 (d, *J* = 8.5 Hz, 2H), 8.29 (d, *J* = 8.6 Hz, 2H), 8.46 (d, *J* = 8.4 Hz, 2H), 8.71 (d, *J* = 4.3 Hz, 1H), 9.13 (d, *J* = 8.2 Hz, 2H). ^13^C NMR (DMSO-d6, 126 MHz): 120.41, 120.77, 122.71, 123.24, 124.28, 125.50, 126.83, 127.22, 127.37, 130.69, 131.72, 132.35, 137.78, 139.06, 143.92, 144.52, 147.53, 149.44, 150.11, 151.36, 155.93, 167.45.

### Detection of singlet oxygen (^1^O_2_)

The ^1^O_2_ generation of the Ir complexes exposed to white light (400–700 nm) irradiation at 50 mW/cm^2^ was evaluated by measuring the absorbance changes of ABDA at 378 nm (pH 5.0) and 380 nm (pH 7.4). In details, ABDA (100 μM) was added to different pH (7.4 and 5.0) of Ir-1 (10 μM) and Ir-2 solutions (10 μM). The absorbance of ABDA in [Ru(bpy)_3_]Cl_2_ was set as a standard, in which ^1^O_2_ quantum yield (*Φ*_Δ_) is 0.18 in air-saturated water [30]. Thereafter, the aforementioned solutions were exposed to white light. The ^1^O_2_ generation of the Ir complex was evaluated by UV–vis spectroscopy at a preset time point. The *Φ*_Δ_ values were calculated according to Eq. (1),

1 $${\Phi_{\Delta (\text{x})}} = {\Phi_{\Delta (\text{std})}} \times \frac{{S_x}}{{S_{std}}}\times \frac{{F_{std}}}{{F_x}}$$

where the subscripts x and std designate the sample and the standard reference, respectively. S is the decomposition rate of ABDA at 378 nm (pH 5.0) and 380 nm (pH 7.4). F is the correction factor of absorption, which is given by F = 1 − 10^−OD^ (OD means the optical density of the sample and [Ru(bpy)_3_]Cl_2_ at 405 nm).

### Preparation and characterization of Ir-NPs

In brief, 2 mg Ir-1 and 10 mg DSPE-mPEG_2000_ were mixed and dissolved in DMF (0.5 mL). Deionized water (1 mL) was then added dropwise to the mixture to prepare the nanoparticles (Ir-NPs). Complete addition was achieved in 1 min. The suspension was maintained under magnetic stirring at 600 rpm for 20 min. All the operations were conducted at room temperature [42]. The nanoparticles were purified by dialysis (*M*_W_ = 3500 Da). The morphologies, elemental mapping, zeta potential, and particle size of Ir-NPs were identified by TEM and DLS. Optical absorption and fluorescence emission spectra were measured using Varian Cary 300 spectrophotometer and Edinburgh FLS 920 spectrometer, respectively.

### Cell line and animal

Skov3 ovarian tumor cells were maintained in RPIM 1640, supplemented with 10% FBS, 100 μg/mL streptomycin and 100 U/mL penicillin. The living cells were cultured in a humidified incubator which provided an atmosphere of 5.0% CO_2_ at 37 °C.

Female BALB/c nude mice and KM mice aged 4–6 weeks were purchased from the animal experiment center of Southern Medical University at Guangzhou. All animal experiments were performed under the guidelines evaluated and approved by the ethics committee of Zhujiang Hospital Southern Medical University, China.

### One/two-photon cellular bioimaging and colocalization assay

After incubating Skov3 cells with Ir-NP (10 μM) for 6 h, MTG (100 nM) was added, and the Skov3 cells were then incubated for another 15 min. After that, the living cells were washed with PBS for three times, and visualized by a CLSM immediately. For the one- and two-photon images of Ir-NPs, the laser with excitation wavelength at 405 nm and 810 nm was adopted, respectively. The excitation wavelength of MTG was 488 nm. Emission was collected at 590 ± 20 nm for Ir-NPs, and 510 ± 20 nm for MTG, respectively.

### Cell internalization assay

For CLSM, Skov3 cells (5 × 10^5^) were seeded in 6-well plates and cultured for 12 h. After treated with Ir-NPs (10 μM) at 37 °C for 2, 4, and 6 h, the cells were fixed with 4% (W/V) paraformaldehyde for 15 min. Then, the nuclei of the cells were stained using DAPI (2 μg/mL, blue) for 5 min. At last, images were collected with CLSM. Emission was collected at 440 ± 20 nm for DAPI and 590 ± 200 nm for Ir-NPs upon excitation at 405 nm. For flow cytometry, after treated with Ir-NPs (10 μM) for 2, 4, and 6 h, the cells were collected and resuspended with PBS for detection.

### Ir-NPs uptake inhibition by various inhibitors

Skov3 cells were seeded in 6-well plates with a density of 5 × 10^5^ cells and cultured for 12 h. After treated with chlorpromazine (CPZ, 30 μM), ethylisopropylamiloride (EIPA, 50 μM), methyl-β-cyclodextrin (Mβ-CD, 5 mM) and filipin III (5 μg/mL), respectively, for 30 min, the cells were incubated with Ir-NPs (10 μM) for another 4 h at 37 °C. The uptake of Ir-NPs with no inhibitor at 37 °C was set as a positive control (4 °C as a negative control). The cellular uptake images were collected with CLSM, then the levels of uptake inhibition were analyzed semiquantitatively using Image J soft.

### Cell viability assay

Skov3 cells (5 × 10^3^) were seeded in 96-well plates and cultured for 12 h. After incubated with Ir-1 and Ir-NPs at various concentrations ranging (from 0.125 to 30 μM) for 12 h, cells were treated with or without white light (400–700 nm) irradiation at 50 mW/cm^2^ for 5 min. The cells were then incubated for 12 h following the standard MTT method.

### Cell death assay

For flow cytometry, Skov3 cells were seeded in 6-well plates and cultured for 12 h, then Ir-1 (2.0 μM) and Ir-NPs (2.0 μM) were added respectively. After 12 h incubation, the cells were treated with or without white light (400–700 nm) irradiation at 50 mW/cm^2^ for 5 min. Thereafter, cells in each group were collected and stained using the Annexin V-FITC/PI apoptosis detection kit according to the procedure given by the manufacture.

For confocal microscopy, Skov3 cells were treated and irradiated as above. Thereafter, cells were washed twice with PBS and incubated with Calcein AM (2 μM)/PI (8 μM) buffer at room temperature for 30 min under dark conditions, then visualized by confocal microscopy immediately.

### Intracellular detection of ROS generation

DCFH-DA was used to evaluate intracellular ROS production. Skov3 were seeded in 6-well plates and incubated for 12 h, after which the cells were treated with Ir-NPs (2.0 μM) for 6 h and following white light (400–700 nm) irradiation at 50 mW/cm^2^ for 5 min. The DCFH-DA (10 μM) was added into each well and incubated for another 20 min. The cells were stained with Hoechst 33342 (10 μg/mL) before fluorescence microscope observation. Moreover, flow cytometry was used to collect the quantitative data for the intracellular ROS level.

### Mitochondrial membrane potential (MMP) detection

JC-1 was used as an indicator of MMP. Skov3 cells were seeded in 6-well plates for 12 h and incubated with Ir-1 (2.0 μM) and Ir-NPs (2.0 μM) for 6 h. After treated with or without white light (400–700 nm) irradiation at 50 mW/cm^2^ for 5 min, the cells were continually incubated for 6 h. Then the cells were collected, stained with JC-1 (5 μg/mL) for 15 min at 37 °C and analyzed by flow cytometry immediately.

### Western blot analysis

Skov3 cells were seeded in 6-well plates and cultured for 12 h. After incubated with Ir-1 (2.0 μM) and Ir-NPs (2.0 μM) for 12 h, cells were treated with or without white light (400–700 nm) irradiation at 50 mW/cm^2^ for 5 min. After 12 h of incubation, the cells were collected to extract protein with radioimmunoprecipitation assay lysis buffer. Equal amounts of these proteins, as determined by BCA Protein Assay Kit, were added to SDS-PAGE gels and separated by gel electrophoresis, respectively. After transferring protein from the gel to the polyvinylidene difluoride (PVDF) membrane, the membrane was blocked with 5% BSA, then incubated with primary antibodies (Bcl-2, Bax, Cytochrome *c* and Caspase-3) and Anti-*β*-actin rabbit monoclonal antibody. Subsequently, the membrane was incubated with goat anti-rabbit IgG antibody. The blots were exposed by an image analysis system (Bio-rad, USA).

### Biodistribution of NPs

Biodistribution was examined in female BALB/c nude mice bearing Skov3 cells. Cy5.5-loaded DSPE-mPEG_2000_ nanoparticles (Cy5.5 NPs) were prepared. Free Cy5.5 and Cy5.5 NPs were intravenously injected into the mice, respectively. An IVIS Lumina III imaging system (E_ex_ = 640 nm, E_em_ = 670 nm) was then used to acquire fluorescence imaging at 0, 2, 4, 8, 12, 24, and 48 h post-injection. Meanwhile, the mice were sacrificed and tumors, as well as the heart, liver, spleen, lung, and kidney were collected for ex vivo fluorescence imaging at 24 h post-injection.

### In vitro imaging of tumor tissue

Ir-NPs in saline (300 μL) were injected via the tail vein of mice. Mice were sacrificed and the tumor was harvested at 24 h post-injection. The tumor high resolution images were recorded with a two-photon fluorescence scanning microscope (E_ex_ = 810 nm, E_em_ = 590 ± 20 nm).

### Hemolysis test

The hemocompatibility level of Ir-1 and Ir-NPs were determined according to the established standard (ISO10993-4). Briefly, the fresh mice blood was obtained from 4 to 6 weeks female KM mice. Subsequently, it was diluted by PBS, and then RBCs were isolated from plasma. After careful washing and dilution, the suspension of RBCs at a final concentration of 2% (*V/V*) was added to Ir-1 (10 μM) and Ir-NPs (10 μM) solution, then incubated at 37 °C in a thermostatic water bath for 3 h. PBS and Triton X-100 (10 g/mL, a surfactant known to lyse RBCs) were used as negative and positive controls, respectively. After RBCs were centrifuged, 100 μL of the supernatant of each sample was transferred to a 96-well plate. The free hemoglobin in the supernatant was measured with a microplate reader at 540 nm. The hemolysis ratio of RBCs was calculated using Eq. (2).

2 $$\mathrm{Hemolytic\,ratio }(\mathrm{\%}) =\frac{{A}_{sample}{-A}_{negative\,control}}{{A}_{positive\, control}{-A}_{negative\,control}}\times 100{\%}.$$

where A_sample_, A_negative control_ and A_positive control_ were denoted as the absorbance of sample, negative and positive controls, respectively.

### Photodynamic therapy and safety evaluation in vivo

The nude mice were assigned to three groups randomly and administrated with PBS, Ir-NPs (0.15 mg/kg) and Ir-NPs (0.15 mg/kg) with white light (400–700 nm) irradiation at 200 mW/cm^2^ for 5 min at 24 h post-injection. The body weight and tumor volume of mice were monitored daily for 3 weeks. The mice were then sacrificed and tumors were harvested for H&E and immunohistochemical staining which was done by Servicebio Biological Technology.

### Statistical analysis

Differences among samples were calculated with the two-tailed Student’s *t*-test using an independent samples *t*-test in SPSS 16.0. Differences among groups were considered statistically significant at P < 0.05.

## Supplementary Information

**Additional file 1.** Additional information includes additional figures.

## Data Availability

All data generated or analyzed during this study are included in this published article and the Additional Information.

## Abbreviations

PDT: Photodynamic therapy
ROS: Reactive oxygen species
Ir: Iridium
AIE: Aggregation-induced emission
NPs: Nanoparticles
PSs: Photosensitizers
^1^O_2_: Singlet oxygen
OH^·^: Hydroxyl radicals
^3^O_2_: Ground-state O_2_
NIR: Near-infrared
UV–vis: Ultraviolet visible
ABDA: 9,10-Anthracenediylbis(methylene)-dimalonic acid
DLS: Dynamic light scatting
TEM: Transmission electron microscopy
EPR: Enhanced permeability and retention
OPE: One-photon excitation
TPE: Two-photon excitation
GLSM: Confocal laser scanning microscope
CPZ: Chlorpromazine
EIPA: Ethylisopropylamiloride
Mβ-CD: Methyl-β-cyclodextrin
MTG: Mito-tracker Green
MMP: Mitochondrial membrane potential
DCFH-DA: 2ʹ,7ʹ-Dichlorofluorescin diacetate
RBCs: Red blood cells
MTT: Diphenyl-2-H-tetrazolium bromide
DAPI: 4ʹ,6-Diamidino-2-phenylindole
BCA: Bicinchoninic acid
PDVF: Polyvinylidene difluoride

## References

[1] Castano AP, Mroz P, Hamblin MR (2006). Photodynamic therapy and anti-tumour immunity. Nat Rev Cancer.

[2] Lucky SS, Soo KC, Zhang Y (2015). Nanoparticles in photodynamic therapy. Chem Rev.

[3] Dolmans DE, Fukumura D, Jain RK (2003). Photodynamic therapy for cancer. Nat Rev Cancer.

[4] Zheng Z, Zhang T, Liu H, Chen Y, Kwok RTK, Ma C, Zhang P, Sung HHY, Williams ID, Lam JWY (2018). Bright near-infrared aggregation-induced emission luminogens with strong two-photon absorption, excellent organelle specificity, and efficient photodynamic therapy potential. ACS Nano.

[5] Hao Y, Chen Y, He X, Yu Y, Han R, Li Y, Yang C, Hu D, Qian Z (2020). Polymeric nanoparticles with ROS-responsive prodrug and platinum nanozyme for enhanced chemophotodynamic therapy of colon cancer. Adv Sci (Weinh).

[6] Lovell JF, Liu TW, Chen J, Zheng G (2010). Activatable photosensitizers for imaging and therapy. Chem Rev.

[7] Cheng L, Wang C, Feng L, Yang K, Liu Z (2014). Functional nanomaterials for phototherapies of cancer. Chem Rev.

[8] Winter A, Schubert US (2016). Synthesis and characterization of metallo-supramolecular polymers. Chem Soc Rev.

[9] Park SY, Oh KT, Oh YT, Oh NM, Youn YS, Lee ES (2012). An artificial photosensitizer drug network for mitochondria-selective photodynamic therapy. Chem Commun (Camb).

[10] Kuang S, Sun L, Zhang X, Liao X, Rees TW, Zeng L, Chen Y, Zhang X, Ji L, Chao H (2020). A mitochondrion-localized two-photon photosensitizer generating carbon radicals against hypoxic tumors. Angew Chem Int Ed Engl.

[11] Wang K-N, Qi G, Chu H, Chao X-J, Liu L-Y, Li G, Cao Q, Mao Z-W, Liu B (2020). Probing cell membrane damage using a molecular rotor probe with membrane-to-nucleus translocation. Mater Horiz.

[12] Kim S, Ohulchanskyy TY, Pudavar HE, Pandey RK, Prasad PN (2007). Organically modified silica nanoparticles co-encapsulating photosensitizing drug and aggregation-enhanced two-photon absorbing fluorescent dye aggregates for two-photon photodynamic therapy. J Am Chem Soc.

[13] Luo J, Xie Z, Lam JW, Cheng L, Chen H, Qiu C, Kwok HS, Zhan X, Liu Y, Zhu D, Tang BZ. Aggregation-induced emission of 1-methyl-1,2,3,4,5-pentaphenylsilole. Chem Commun (Camb). 2001:1740–1. 10.1039/b105159h 12240292

[14] Feng G, Liu B (2018). Aggregation-induced emission (AIE) dots: emerging theranostic nanolights. Acc Chem Res.

[15] Wu W, Mao D, Hu F, Xu S, Chen C, Zhang CJ, Cheng X, Yuan Y, Ding D, Kong D, Liu B (2017). A highly efficient and photostable photosensitizer with near-infrared aggregation-induced emission for image-guided photodynamic anticancer therapy. Adv Mater.

[16] Li Q, Li Y, Min T, Gong J, Du L, Phillips DL, Liu J, Lam JWY, Sung HHY, Williams ID (2020). Time-dependent photodynamic therapy for multiple targets: a highly efficient AIE-active photosensitizer for selective bacterial elimination and cancer cell ablation. Angew Chem Int Ed Engl.

[17] Wang D, Zhu L, Pu Y, Wang JX, Chen JF, Dai L (2017). Transferrin-coated magnetic upconversion nanoparticles for efficient photodynamic therapy with near-infrared irradiation and luminescence bioimaging. Nanoscale.

[18] Sun L, Ge X, Liu J, Qiu Y, Wei Z, Tian B, Shi L (2014). Multifunctional nanomesoporous materials with upconversion (in vivo) and downconversion (in vitro) luminescence imaging based on mesoporous capping UCNPs and linking lanthanide complexes. Nanoscale.

[19] Zhou Z, Song J, Nie L, Chen X (2016). Reactive oxygen species generating systems meeting challenges of photodynamic cancer therapy. Chem Soc Rev.

[20] Liu Z, Romero-Canelón I, Qamar B, Hearn JM, Habtemariam A, Barry NP, Pizarro AM, Clarkson GJ, Sadler PJ (2014). The potent oxidant anticancer activity of organoiridium catalysts. Angew Chem Int Ed Engl.

[21] Feng L, Geisselbrecht Y, Blanck S, Wilbuer A, Atilla-Gokcumen GE, Filippakopoulos P, Kräling K, Celik MA, Harms K, Maksimoska J (2011). Structurally sophisticated octahedral metal complexes as highly selective protein kinase inhibitors. J Am Chem Soc.

[22] Wilbuer A, Vlecken DH, Schmitz DJ, Kräling K, Harms K, Bagowski CP, Meggers E (2010). Iridium complex with antiangiogenic properties. Angew Chem Int Ed Engl.

[23] Lo KK (2015). Luminescent Rhenium(I) and Iridium(III) polypyridine complexes as biological probes, imaging reagents, and photocytotoxic agents. Acc Chem Res.

[24] You Y (2013). Phosphorescence bioimaging using cyclometalated Ir(III) complexes. Curr Opin Chem Biol.

[25] Zhao Q, Huang C, Li F (2011). Phosphorescent heavy-metal complexes for bioimaging. Chem Soc Rev.

[26] Woo H, Cho S, Han Y, Chae WS, Ahn DR, You Y, Nam W (2013). Synthetic control over photoinduced electron transfer in phosphorescence zinc sensors. J Am Chem Soc.

[27] Wang KN, Liu LY, Qi G, Chao XJ, Ma W, Yu Z, Pan Q, Mao ZW, Liu B (2021). Light-driven cascade mitochondria-to-nucleus photosensitization in cancer cell ablation. Adv Sci (Weinh).

[28] Cao R, Jia J, Ma X, Zhou M, Fei H (2013). Membrane localized iridium(III) complex induces endoplasmic reticulum stress and mitochondria-mediated apoptosis in human cancer cells. J Med Chem.

[29] Ma DL, Chan DS, Leung CH (2014). Group 9 organometallic compounds for therapeutic and bioanalytical applications. Acc Chem Res.

[30] Houten JV, Watts RJ (1976). Temperature dependence of the photophysical and photochemical properties of the tris(2,2'-bipyridyl)ruthenium(II) ion in aqueous solution. J Am Chem Soc.

[31] Legrand P, Lesieur S, Bochot A, Gref R, Raatjes W, Barratt G, Vauthier C (2007). Influence of polymer behaviour in organic solution on the production of polylactide nanoparticles by nanoprecipitation. Int J Pharm.

[32] Wang J, Zhou J, He H, Wu D, Du X, Xu B (2019). Cell-compatible nanoprobes for imaging intracellular phosphatase activities. ChemBioChem.

[33] Wang B, Van Herck S, Chen Y, Bai X, Zhong Z, Deswarte K, Lambrecht BN, Sanders NN, Lienenklaus S, Scheeren HW (2020). Potent and prolonged innate immune activation by enzyme-responsive imidazoquinoline TLR7/8 agonist prodrug vesicles. J Am Chem Soc.

[34] Son JM, Lee C (2021). Aging: all roads lead to mitochondria. Semin Cell Dev Biol.

[35] Jiang X, Jiang H, Shen Z, Wang X (2014). Activation of mitochondrial protease OMA1 by Bax and Bak promotes cytochrome c release during apoptosis. Proc Natl Acad Sci USA.

[36] Desagher S, Martinou JC (2000). Mitochondria as the central control point of apoptosis. Trends Cell Biol.

[37] Smiley ST, Reers M, Mottola-Hartshorn C, Lin M, Chen A, Smith TW, Steele GD, Chen LB (1991). Intracellular heterogeneity in mitochondrial membrane potentials revealed by a J-aggregate-forming lipophilic cation JC-1. Proc Natl Acad Sci USA.

[38] Ly JD, Grubb DR, Lawen A (2003). The mitochondrial membrane potential (deltapsi(m)) in apoptosis; an update. Apoptosis.

[39] Zhang C, Wu J, Liu W, Zheng X, Zhang W, Lee C-S, Wang P (2020). Hypocrellin-based multifunctional phototheranostic agent for NIR-triggered targeted chemo/photodynamic/photothermal synergistic therapy against glioblastoma. ACS Appl Bio Mater.

[40] Jiang L, Zhou Q, Mu K, Xie H, Zhu Y, Zhu W, Zhao Y, Xu H, Yang X (2013). pH/temperature sensitive magnetic nanogels conjugated with Cy5.5-labled lactoferrin for MR and fluorescence imaging of glioma in rats. Biomaterials.

[41] Liu J, Jin C, Yuan B, Liu X, Chen Y, Ji L, Chao H (2017). Selectively lighting up two-photon photodynamic activity in mitochondria with AIE-active iridium(iii) complexes. Chem Commun (Camb).

[42] Chen Q, Yang Y, Lin X, Ma W, Chen G, Li W, Wang X, Yu Z (2018). Platinum(iv) prodrugs with long lipid chains for drug delivery and overcoming cisplatin resistance. Chem Commun (Camb).

