# Supplementary material for: Multifunctional AIE iridium (III) photosensitizer nanoparticles for two-photon-activated imaging and mitochondria targeting photodynamic therapy
Source: J Nanobiotechnology. 2021 Aug 23;19:254. doi: 10.1186/s12951-021-01001-4 (PMC8381541; doi:10.1186/s12951-021-01001-4)
Supplement: Supplementary file 1 — Additional file 1. Additional information includes additional figures. [file 12951_2021_1001_MOESM1_ESM.docx]

**Multifunctional AIE Iridium (III) Photosensitizer Nanoparticles for Two-Photon-Activated Imaging and Mitochondria Targeting Photodynamic Therapy**

Xuzi Cai^1†^, Kang-Nan Wang^2, 3†^, Wen Ma^4^, Yuanyuan Yang^4^, Gui Chen^4^, Huijiao Fu^1^, Chunhui Cui^5*^, Zhiqiang Yu^2,4*^, Xuefeng Wang^1*^

^*^ Corresponding author: [douwangxuefeng@163.com](mailto:douwangxuefeng@163.com); yuzq@smu.edu.cn; [drcuich@163.com](mailto:drcuich@163.com)

^†^ Xuzi Cai and Kang-Nan Wang contributed equally

^1^ Department of Obstetrics and Gynecology, The Third Affiliated Hospital of Southern Medical University, Guangzhou 510632, China

^2^ Shunde Hospital, Southern Medical University (The First People's Hospital of Shunde), Foshan, Guangdong 528308, China

^3^ Department of Chemical and Biomolecular Engineering, National University of Singapore, 4 Engineering Drive 4, Singapore 117585, Singapore

^4^ Guangdong Provincial Key Laboratory of New Drug Screening, School of Pharmaceutical Sciences, Southern Medical University, Guangzhou 510515, China

^5^ Department of General Surgery, Zhujiang Hospital of Southern Medical University, Guangzhou 510250, China

Scheme S1. Schematic routes of Ir-1 and Ir-2. (a) CH_3_COOH, reflux overnight [1]. (b) CHCl_3_/CH_3_OH (2:1, v/v), Ar, 65 ℃, dark, 12 h.


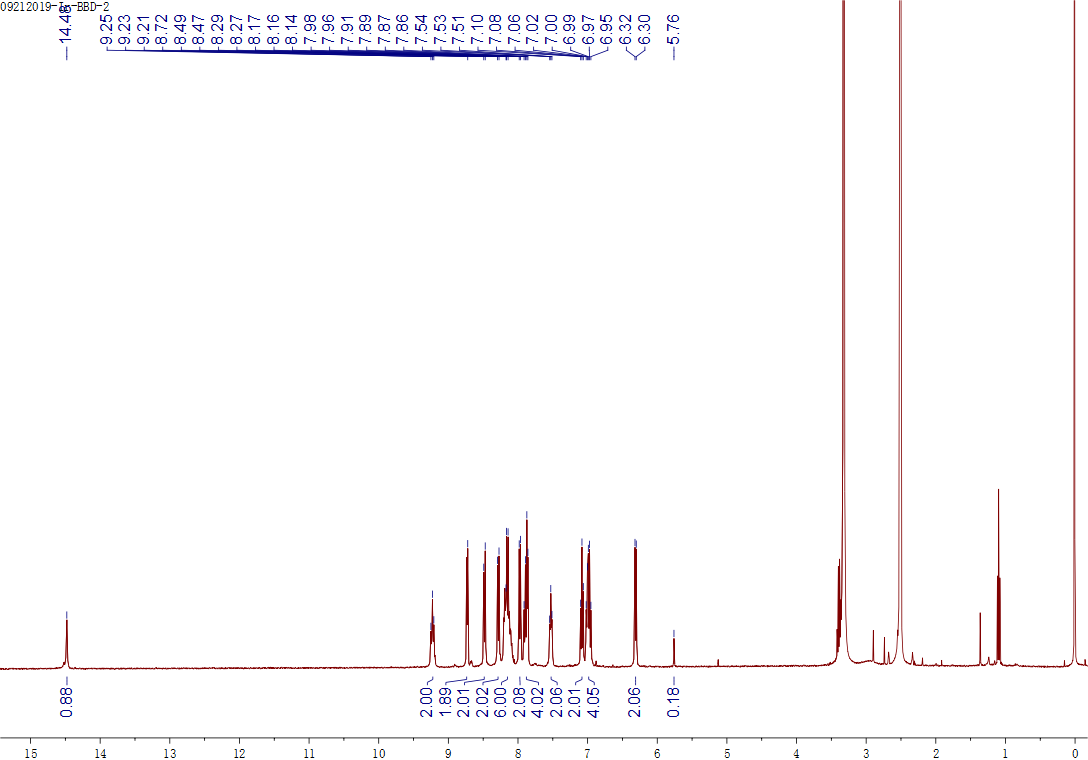


Figure S1. ^1^H NMR spectrum of Ir-1.


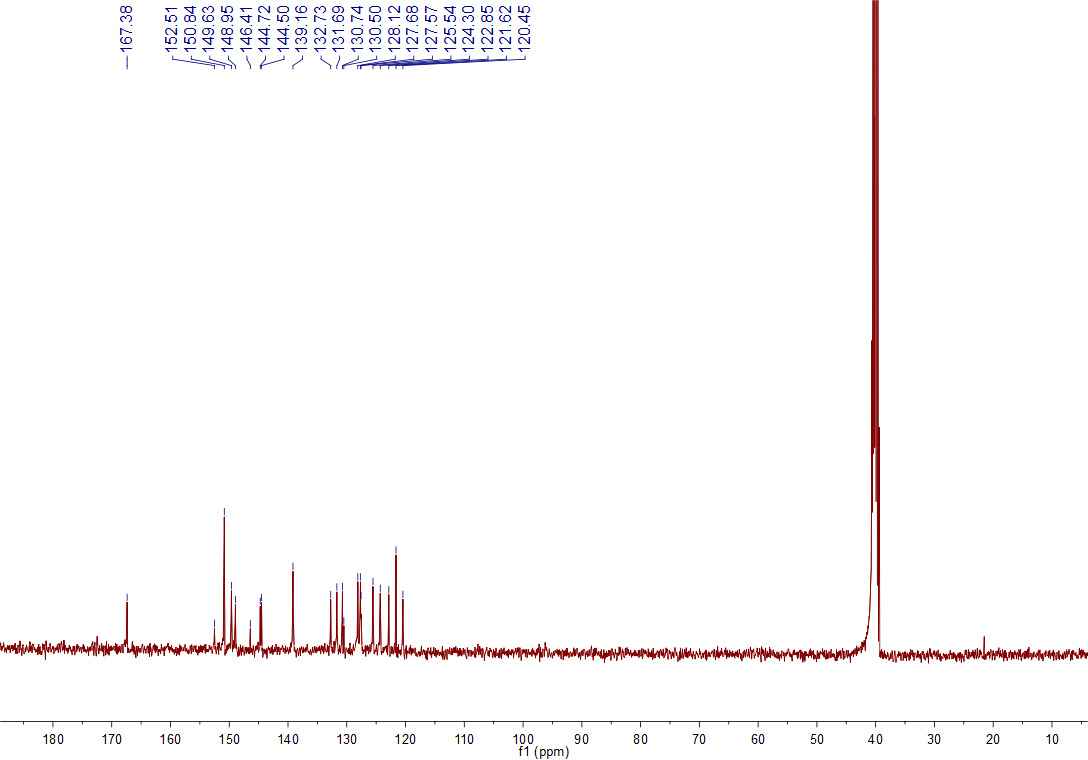


Figure S2. ^13^C NMR spectrum of Ir-1.


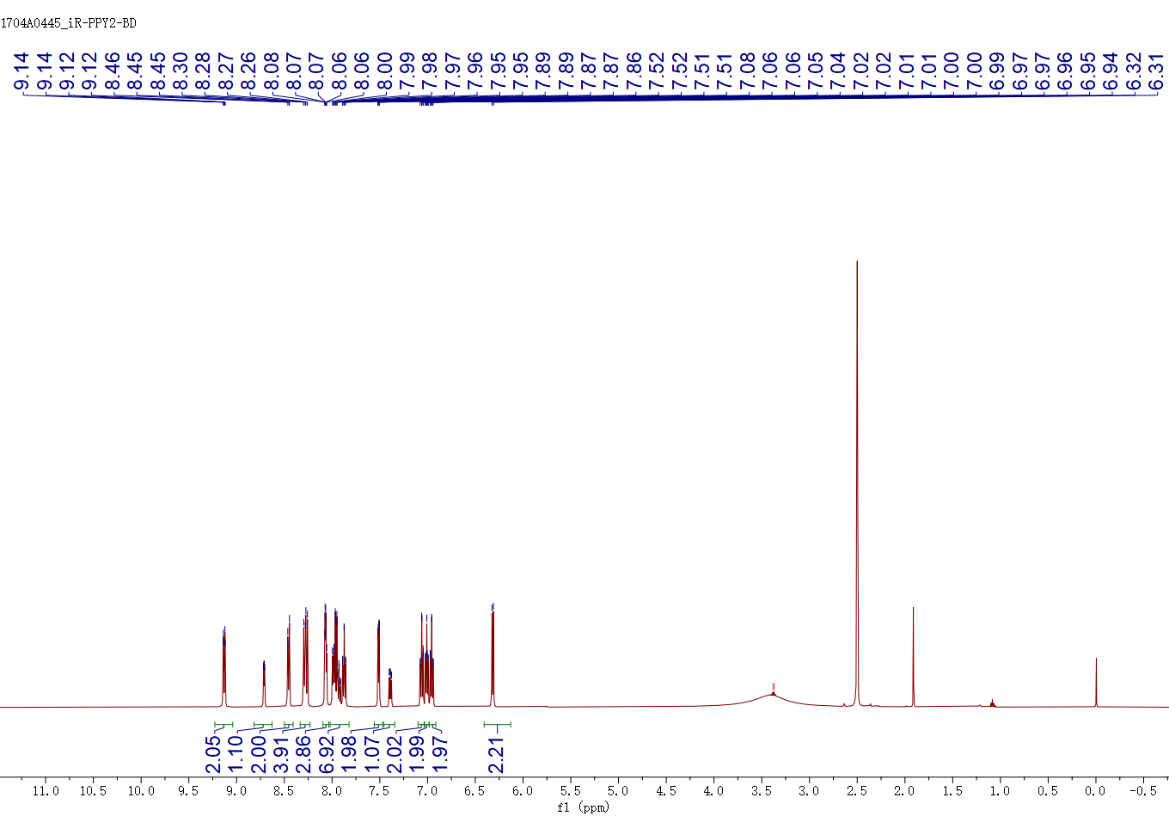


Figure S3. ^1^H NMR spectrum of Ir-2.


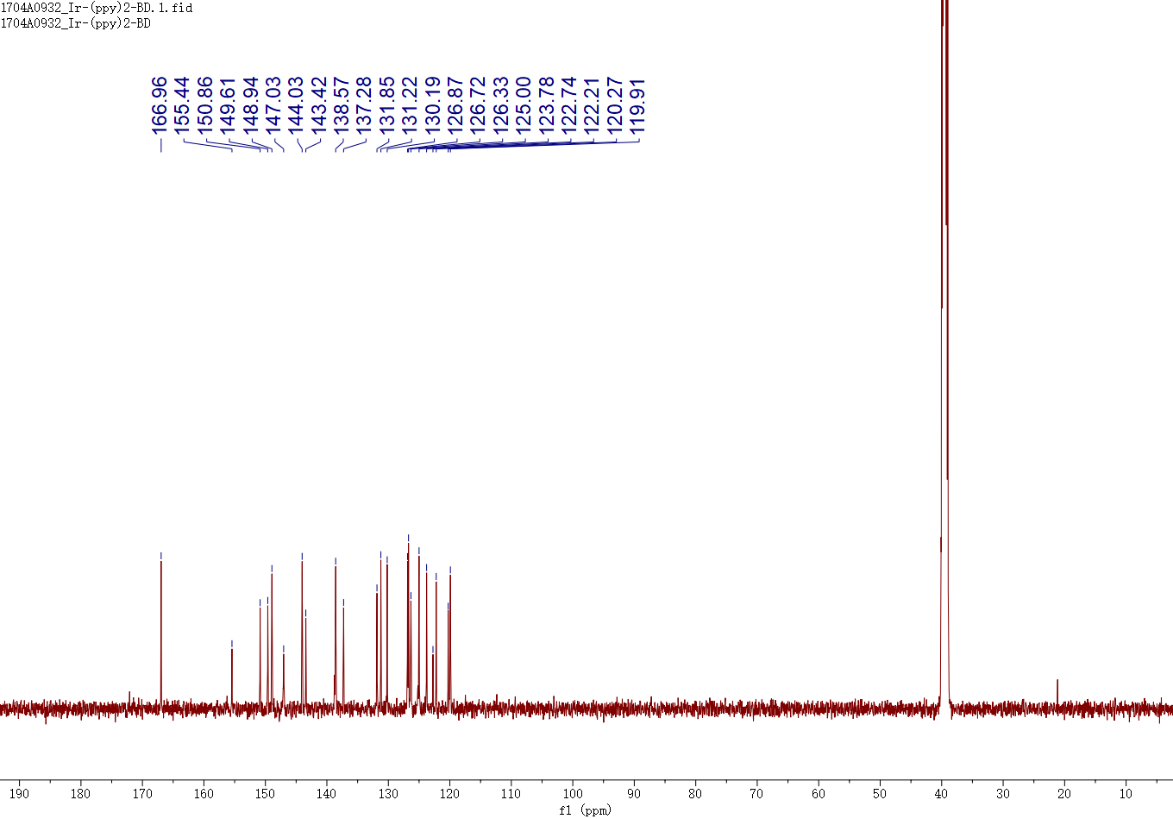


Figure S4. ^13^C NMR spectrum of Ir-2.


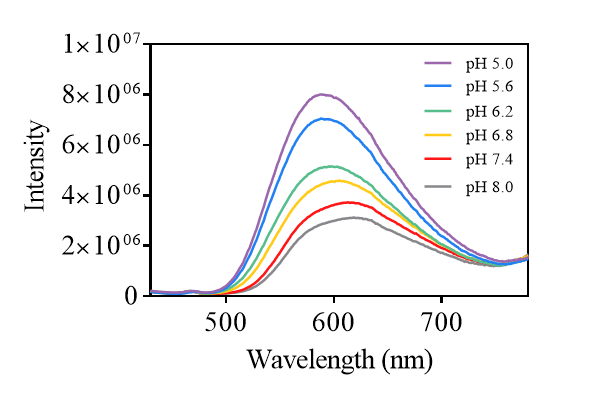


Figure S5. pH-sensitive emission spectra of Ir-2 in disodium hydrogen phosphate-citric acid buffer solution.


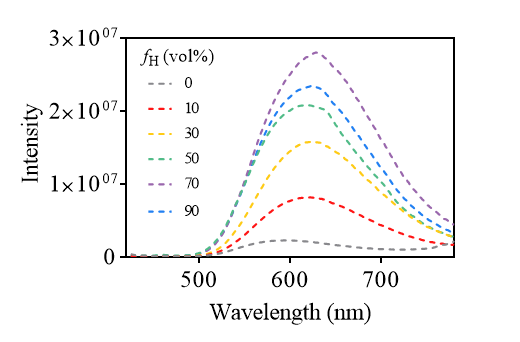


Figure S6. Emission spectra of Ir-2 in H_2_O/DMSO mixtures with different H_2_O fractions (*f*_H_).


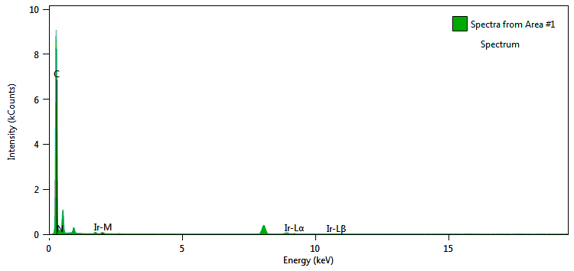


Figure S7. X-ray energy dispersive spectrum of Ir-NPs.


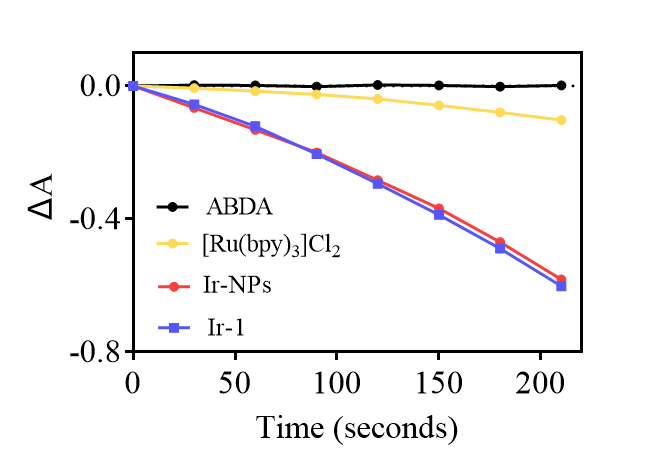


**Figure S8**. The decomposition rate of ABDA (100 μM) for Ir-NP (10 μM) and Ir-1 (10 μM) at pH 7.4 under white light irradiation (50 mW/cm^2^).


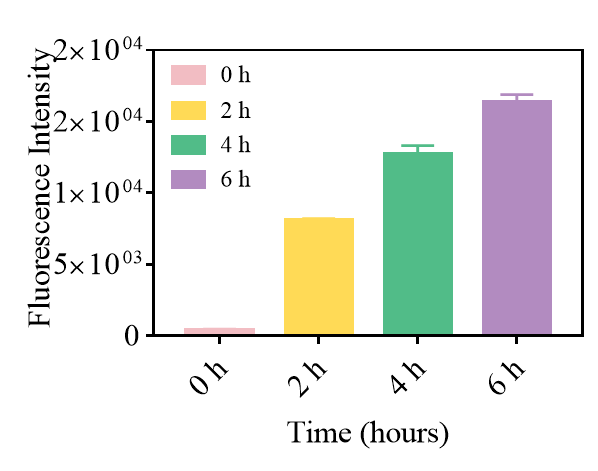


Figure S9. Intracellular Ir-NPs uptake of Skov3 cells tested by flow cytometer for 2, 4, and 6 h.


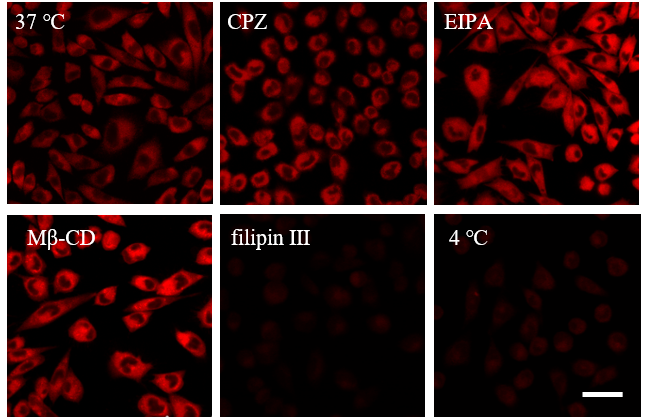


Figure S10. The CLSM images of Ir-NPs uptake by Skov3 cells upon different treatments of endocytosis inhibitors (CPZ, EIPA, Mβ-CD, and filipin Ⅲ) at 37 ℃ and 4 ℃. Scale bar: 50 μm.


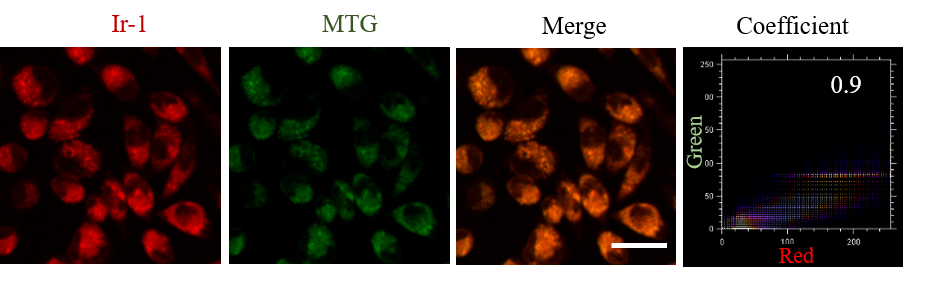


Figure S11. The OPE confocal images of Ir-1 distribution in Skov3 cells. Scale bar: 10 μm.


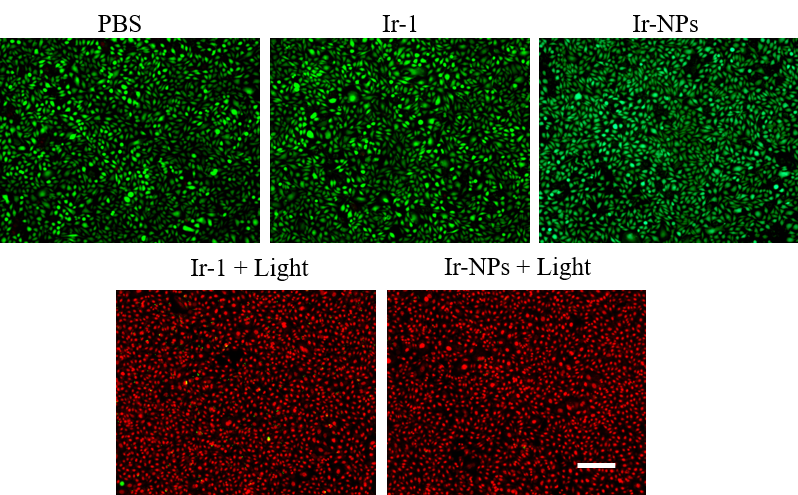


Figure S12. The Calcein-AM/PI staining of Skov3 cells treated with PBS, Ir-1, Ir-NPs, Ir-1 + Light, and Ir-NPs + Light (400-700 nm, 50 mW cm^-2^, 5 min). Scaler bar: 150 μm.


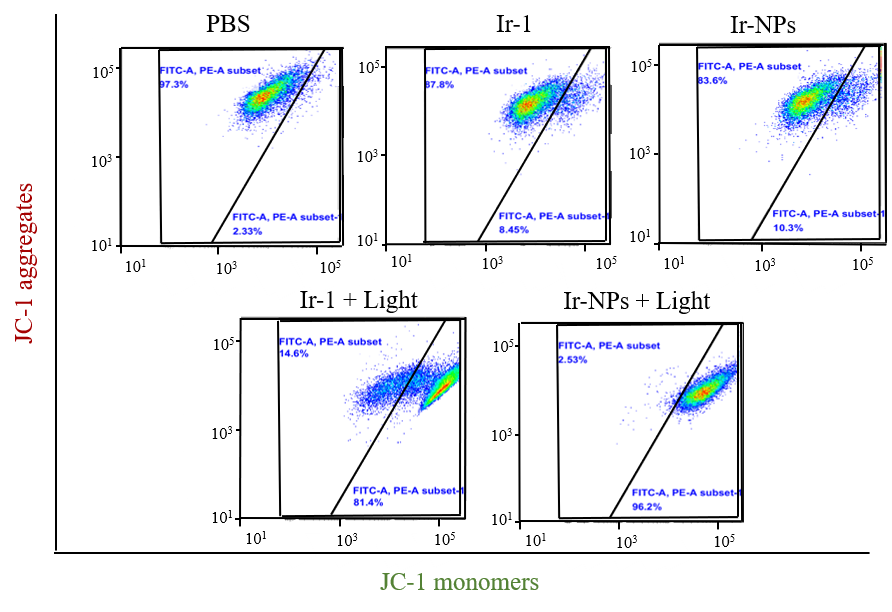


Figure S13. Flow cytometer images of mitochondrial membrane potential (using JC-1 as indicator) for Skov3 cells treated with PBS, Ir-1, Ir-NPs, Ir-1 + Light, Ir-NPs + Light (400-700 nm, 50 mW cm^-2^, 5 min).


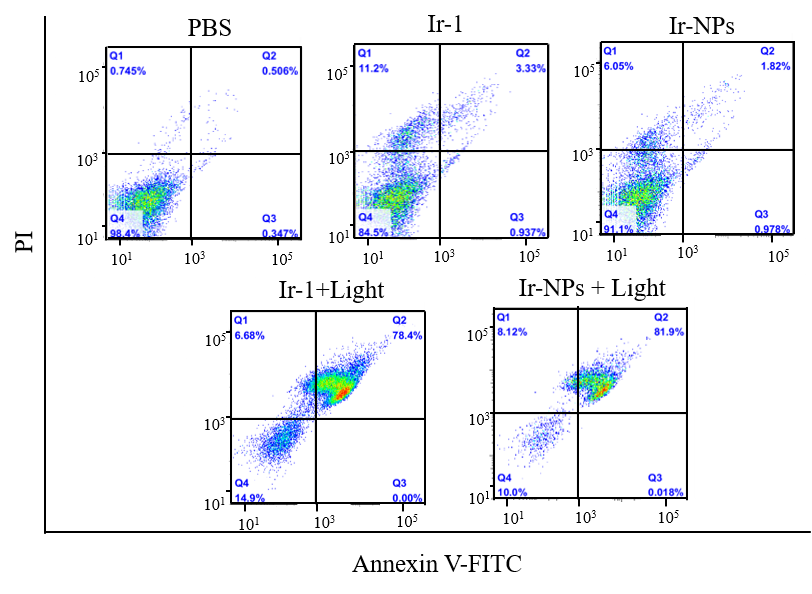


Figure S14. Cell apoptosis images of Skov3 after treatment with PBS, Ir-1, Ir-NPs, Ir-1 + Light, and Ir-NPs + Light (400-700 nm, 50 mW cm^-2^, 5 min).


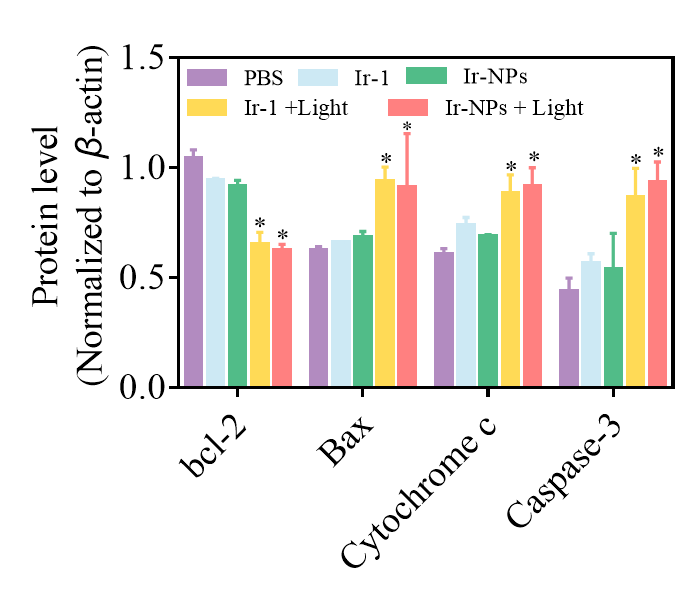


Figure S15. Expression levels of indicated proteins in Skov3 cells were detected using semiquantitative analyses. *β*-actin was used as a control. ^*^*P* < 0.05.


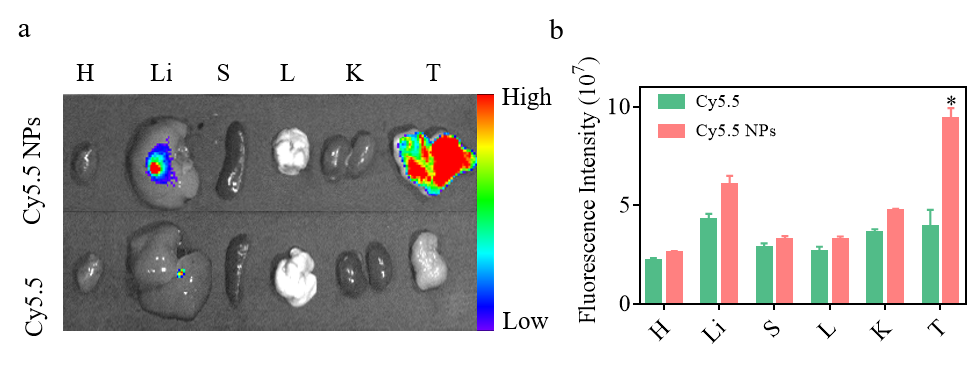


Figure S16. (a) *Ex vivo* fluorescence distribution in tumor and major organs and (b) its corresponding quantification of Cy5.5 and Cy5.5 NPs 24 h post-injection.


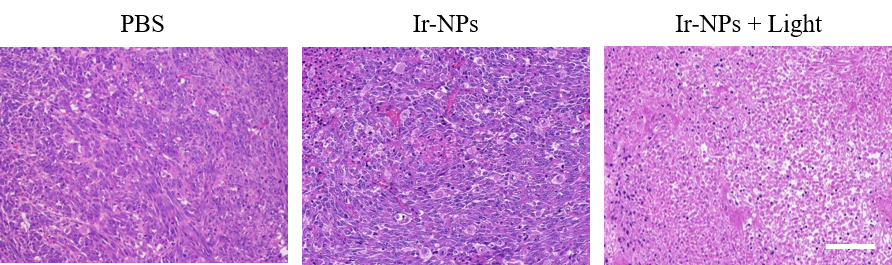


Figure S17. H&E staining of tumors from ovarian cancer model after treatment with PBS, Ir-NPs and Ir-NPs + Light (400-700 nm, 200 mW cm^-2^, 5 min). Scale bar = 100 μm.


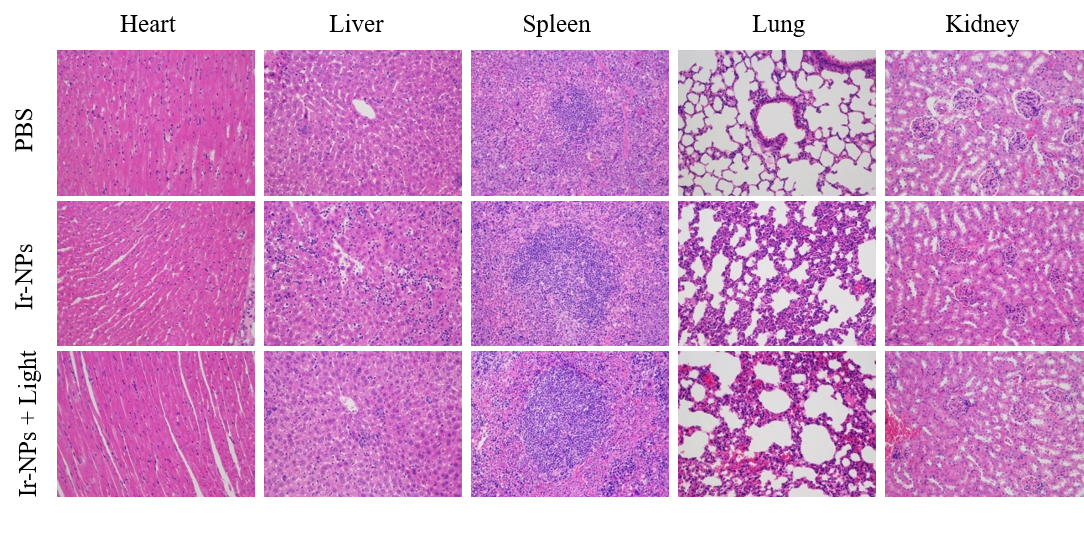


Figure S18. *In vivo* biological safety evaluated by H&E analysis of major organs. Scale bar = 100 μm.

**Reference**

1. Jin C, Liu J, Chen Y, Guan R, Ouyang C, Zhu Y, Ji L, Chao H: **Cyclometalated Iridium(III) Complexes as AIE Phosphorescent Probes for Real-Time Monitoring of Mitophagy in Living Cells.** *Sci Rep* 2016, **6:**22039.
